# Refugees’ Care Experiences, Self-Reported Health Outcomes and Transition to Mainstream Health Care After One Year at the Refugee Engagement and Community Health (REACH) Clinic

**DOI:** 10.1007/s10903-023-01534-w

**Published:** 2023-09-05

**Authors:** Monique Reboe-Benjamin, Mahli Brindamour, Karen Leis, Jacelyn Hanson, Lori Verity-Anderson, Maria Gomez, Melanie Baerg, Anne Leis

**Affiliations:** 1 Department of Community Health and Epidemiology, College of Medicine, University of Saskatchewan, Saskatoon, SK Canada; 2 Department of Pediatrics, College of Medicine, University of Saskatchewan, Saskatoon, SK Canada; 3 Refugee Engagement and Community Health (REACH) Clinic liaison the Saskatoon Community Clinic, Saskatoon, SK Canada; 4 Saskatoon Open Door Society, Saskatoon, SK Canada; 5 Health & Case Coordination at Global Gathering Place, Saskatoon, SK Canada

**Keywords:** Refugee access, Access to care, Transition care, Model of care

## Abstract

This study reports how refugees experienced care at an integrated clinic during their first year in Canada and how they transitioned to a community physician. A survey was completed by 75 Government Assisted Refugees followed at the REACH clinic between 2018 and 2020; 16 agreed to an additional interview. Regression modelling explored the relationship between “perceived health status at one year” and several independent variables. Qualitative thematic analysis provided context. Tailored access to care and enhanced communication through interpretation contributed to satisfaction with clinic services. A significant positive relationship was found between their perceived health status and frequency of visits (p < 0.042), and “doctors’ advice about how to stay healthy” (p < 0.039). Interview findings highlighted the important role of settlement agencies, timing for a successful transition and physicians’ support resources. While refugees benefit from attending integrated clinics, these should also prepare the care transition to community physicians. Targeted government funding and continued medical education could enhance refugees’ transition experience.

## Introduction

Between 2015 and 2020, Canada resettled approximately 154,820 refugees. Of that number, Saskatchewan hosted 5,900. The city of Saskatoon welcomed 2,690 refugees during that period [1, 2]. At the time of this study, refugees in Canada, and by extension Saskatoon, came predominately from countries throughout Africa [particularly, Burundi, Eritrea, Democratic Republic of Sudan, Democratic Republic of Congo] and Asia [3]. In most of these low-middle-income Sub-Saharan African countries, healthcare access is based on a user fee service model. However many are hoping to move to universal health care access [4]. According to published literature, refugees’ health care needs are complex [5, 6] and therefore they require integrated, community-based primary health care interventions in a culturally safe and timely manner [7, 8]. In 2016, the Saskatoon Refugee Health Collaborative^1^ piloted a dedicated primary health care facility to service the city’s growing refugee population. It was transformed in February 2017 into a regular service known as the Refugee Engagement and Community Health Clinic: REACH Clinic, which operated a few half days per week. The primary goal of this community-based initiative is to provide a multi-disciplinary integrated service delivery model which meets refugees’ health needs in one place and contributes to their integration into the health care system. Typically, a transition to a community physician is planned after one year with the assistance of settlement counsellors.

While such models of care for refugees have been described in the literature [9], little is known about refugees’ perceptions of care and health care utilization experiences within such models. Self-reported outcomes and ultimate transition to a community family physician have not been well documented either.

The purpose of this article is to report on refugees’ experiences and self-perceived outcomes while accessing the REACH clinic during the first year following their arrival in Saskatoon, Canada. The transition to mainstream care was also explored from their perspectives.

## Methods

### Study Design

A parallel mixed-methods design was followed to capture quantitatively their experiences of clinic services, and qualitatively their perceptions of the transition to mainstream care. The model of care by Cooper et al. served as a guiding framework to organize the findings [10].

### Participants

To participate, refugees had to be 16 years and older, received one year of care at the REACH, and subsequently graduated from the one-year clinic program. A consecutive sampling strategy ensured all 100 clinic patients who completed the one-year program between March 2017 and February 2018 were informed about the study and evaluated for eligibility.

### Participants’ Characteristics

Following consent, seventy-five government assisted refugees responded to the survey, representing a response rate of 75%. Among the participants, 49% were within the age group 25–44 and 51% were males, as depicted in Table 1. Almost ¾ of the sample originated from African countries, predominately Congo, Burundi, Somalia, and Eritrea, while ¼ came from Asian countries such as Syria and Pakistan.

**Table 1:** Study participants’ characteristics by gender

		Total	
	Overall Total N = 75	Female N = 37	Male N = 38
Characteristics	n(%)	n(%)	n(%)
**Age** 16–24 25–44 45+	15 (21) 35 (49) 22 (30)	6 (18) 19 (56) 9 (26)	9 (24) 16 (42) 13 (34)
**Total**	**72**	**34 (47)**	**38 (53)**
**Education** Primary High school or greater	44 (66) 24 (34)	24 (54) 9 (37)	20 (46) 15 (63)
**Total**	**68**	**33 (48)**	**35 (52)**
Continent* Africa Asia	38 (70) 18 (30)	18 (47) 8 (44)	20 (53) 10 (56)
**Total**	**56**	**26 (46)**	**30 (54)**

*African countries represented were Burundi, Ethiopia, Zimbabwe, Congo, Sudan, Eritrea and Somalia

Asian countries were Syria, Iraq, Pakistan, Iran

### Data Collection

#### Quantitative Component/Survey

An advisory committee comprising settlement agencies, physicians, clinic representatives, and researchers developed the survey. The survey explored participants’ perceptions of care such as access to clinic services, communication and interpretation, role of REACH physicians and clinic impact. Some clinic evaluation questions were formulated by the advisory committee. These were written in simple English for easy comprehension. In addition, the survey included items relating to demographics, health status and health outcomes taken from Statistics Canada Surveys, RAND-36 item health survey [11], and measures of mental health and post-traumatic stress [12, 13]. We piloted the survey with five refugees who were former clinic patients and later revised it for conciseness and clarity. Interpreters administered the survey in the language of comfort for the participants.

#### Qualitative Component/Interview

Nine male and seven female participants from Burundi, Democratic Republic of Congo, Sudan, Eritrea, Pakistan, and Syria agreed to a more in-depth interview. These participants had transitioned to a community physician 9–12 months after completing the initial survey and had accessed mainstream health care. The interviews explored further their clinic care experiences as well as their transition to community health care. The researcher followed a semi-structured interview guide as shown below:

What did you think going to the doctor would be like in Canada?

How do you feel about the care you received at the REACH clinic?

What was your experience at the clinic with your children?

How did you feel about having to change from the doctor at REACH to a new family physician?

What is it like for you now when you need to visit the doctor or use any health service?

An interpreter helped with the communication when needed. Interviews lasted approximately 30–45 min and were audio-recorded if participants consented; otherwise, notes were taken and summarized.

### Analysis

We conducted descriptive statistics for survey responses. Multivariable Logistic Regression analysis assessed the association between ‘health status at one year’ and survey responses which were categorized according to the Cooper et al. framework [10]. The health status variable “compared to one year ago, how would you say your health is now?“ was scored as a five-point Likert scale ranging from “much better than one year ago” to “much worse than one year ago”. The simplest re-coding of this variable created a dichotomous variable of “positive change in health status” and “no change or negative change”. Data was analysed using the SPSS software^2^.

Interviews were analysed thematically using the six-phase approach outlined by Braun and Clarke [14]. We triangulated quantitative and qualitative data during the analysis process, which enabled the authors to connect and interpret both data sets simultaneously through convergence and corroboration.

#### Ethics Approval

This study was approved by the Research Ethics Board of the University of Saskatchewan.

## Results

### Access to Clinic Services

**Fig. 1:**
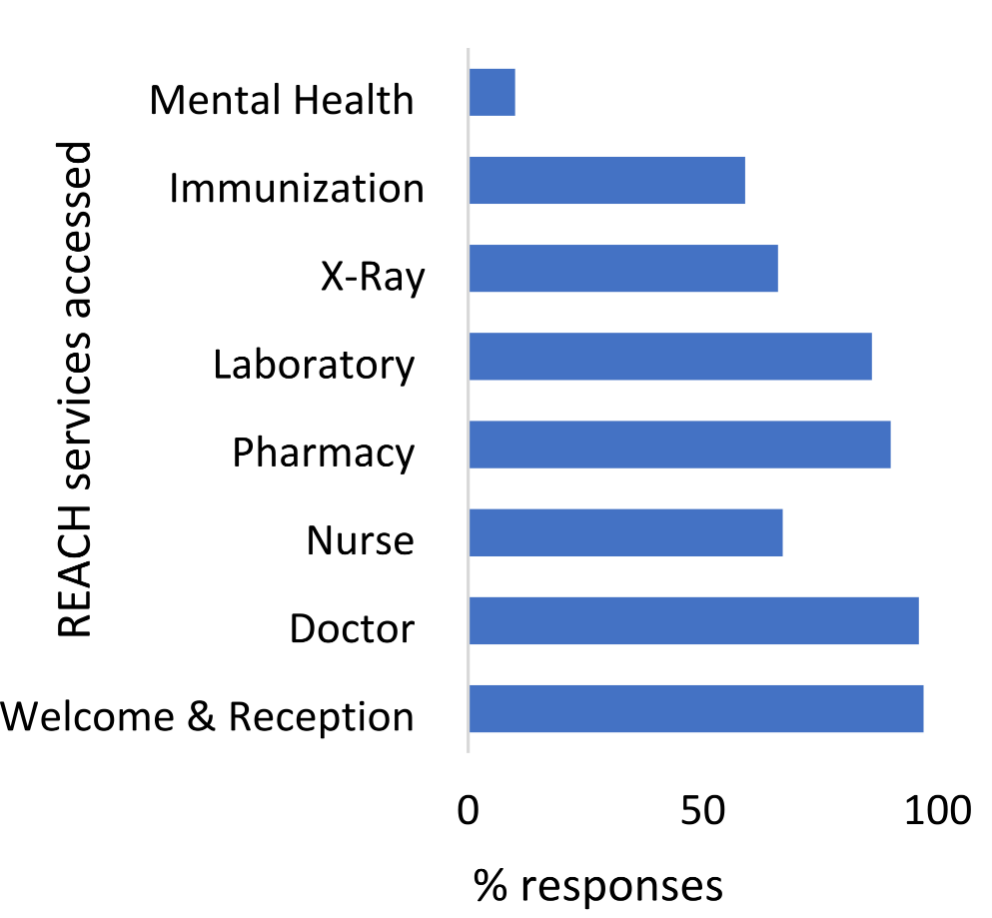
Frequency of utilization of REACH clinic services

**Fig. 2:**
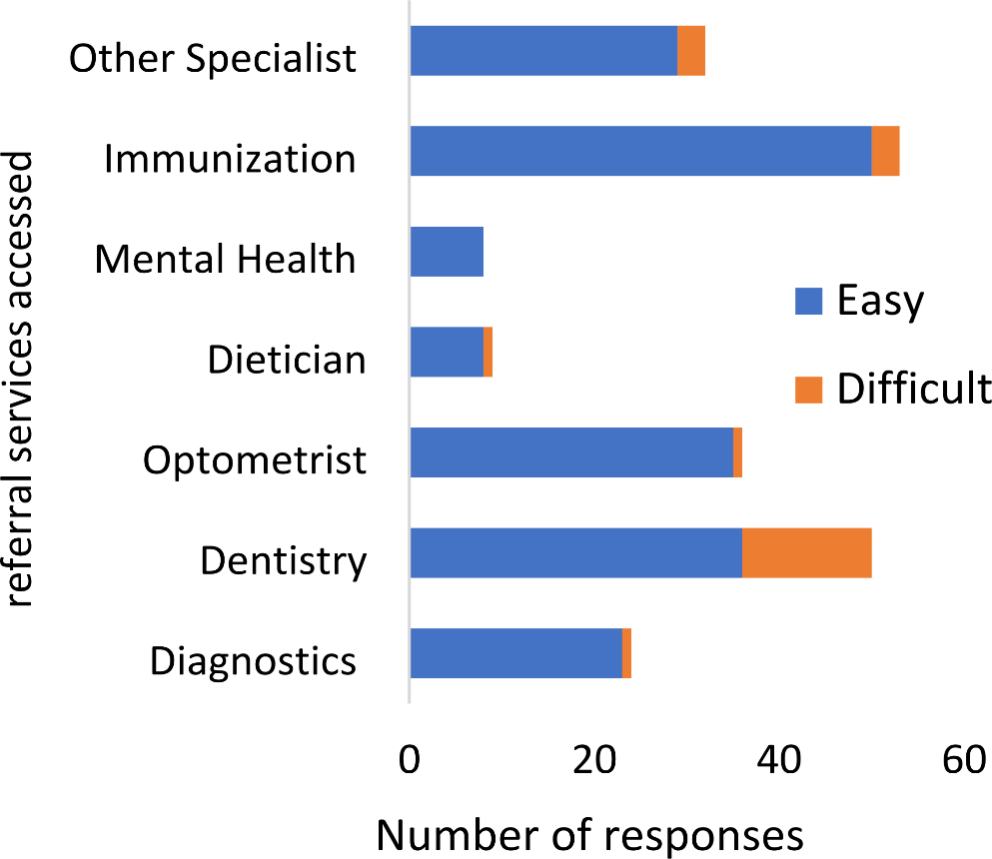
Ease of access of referral services

As shown in Fig. 1, respondents accessed a range of essential services, all located at REACH, such as pharmacy (90%), laboratory (86%) and x-ray (66%) services, including referrals to specialists, which the majority found easier to access through the clinic. Dentistry was the most difficult service to access as seen in Fig. 2, because of cost. Participants unfamiliarity with the transportation services and the winter conditions prevented some from accessing clinic services consistently- “*I don’t know how to get to another lab, I have to wait for someone to take me.” (P015)*

Approximately ¾ of the respondents visited the clinic more than 5 times and reported having access to appointments when needed. Interview findings indicated some reasons for the high usage of services such as being pregnant or having several children in the family. When asked what they appreciated about the clinic, one mother remarked, *“at the clinic it was easy, they have everything that we need if you want to do a blood test, it’s there if you want to go to the pharmacy, it’s there.” (P008)*

### Perceptions of Communication and Interpretation

82% of respondents benefitted from an in-person interpreter, while 67% also used a telephone interpretation service to communicate. Approximately 55% felt interpretation was always available, and 2/3 thought it was very helpful (Table 2). During the interviews, participants expressed that clinic interpreters helped them understand what was occurring medically. “*When we came, we had lots of blood work going – like 10 tubes – but because of the language barrier, we had lots of questions, why doing blood work, we did this before we came. We did not understand. But they explained everything through interpretation. I was glad for that.” (P011)*

**Table 2:** Participants’ perceptions of accessibility, communication, and care appropriateness while at the REACH Clinic

Survey Questions	Responses = n (%)
**Access to clinics services:**	
Were you able to access all your appointments when needed? (N = 73, missing 2)	Yes **=** 55 (75%)
Frequency of visits (N = 74, missing 1)	Up to 4 = 20 (27%) 5 and over = 54 (73%)
**Language and communication: **	
What method of interpretation did you have? (N = 72) In-person Telephone Friends/Family None	Yes = 59 (82%) Yes = 48 (67%) Yes = 10 (14%) Yes = 5 (7%)
Was interpretation available at every appointment? (N = 72, missing 3)	Yes = 38 (53%)
Did you find the interpretation helpful? (N = 65, missing 10)	Helpful = 61 (94%)
Was the in-person interpreter respectful of your values? (N = 63, missing 12)	Yes = 57 (90%)
**Clinic appropriateness:**	
Did the clinic team explain procedures to you in a way you could understand? (N = 73, missing 2)	Yes = 67 (92%)
Were clinic staff/providers friendly and courteous? (N = 72, missing 3)	Yes = 51 (71%)
Were you comfortable with the gender of your doctor? (N = 75, missing 0)	Yes = 64 (85%)
**Transportation**	
How easy was it to get to the clinic? (N = 75, missing 0)	Easy = 43 (57%)

One female participant who sought care outside the clinic mentioned having to resort to her young daughter as an interpreter. After transition participants had limited access to interpretation which resulted in much misunderstanding and some financial consequences.

### Complementary Roles of Physicians and Settlement Councillors

**Fig. 3:**
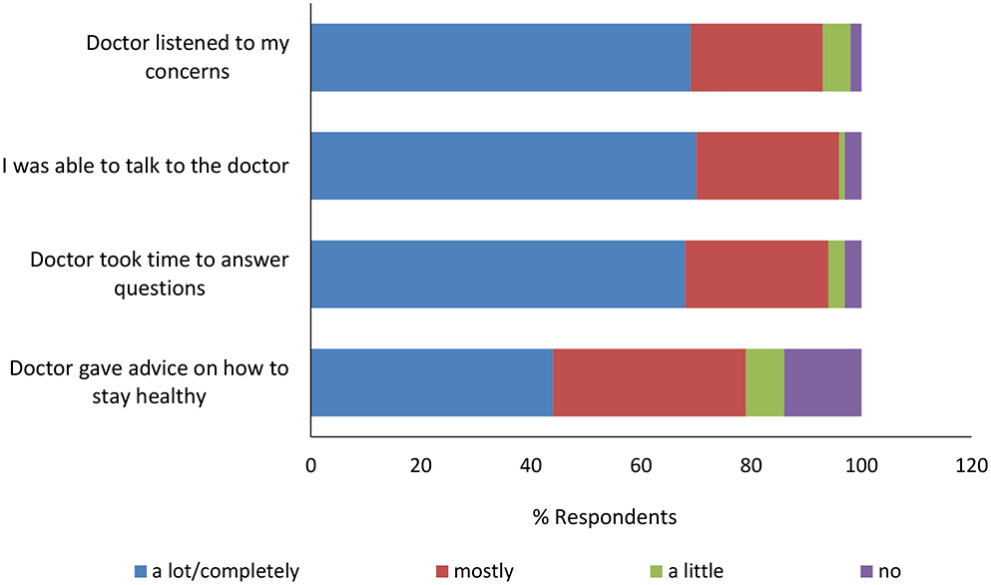
Perception of visit with healthcare provider (N=73)

94% of respondents mostly or completely agreed with the statement: “clinic doctors took time to answer my questions”, and 96% mostly or completely agreed with the statement: “I was able to talk to the physicians about my health concerns”, highlighting the positive provider-patient interactions (Fig. 3). During the interview, several participants reported being impressed with the quality of services their children received.

The clinic has strong ties to the settlement agencies. These effective partnerships contributed significantly to participants’ satisfaction of care, their understanding of health services and other social services around them: *“My counsellors [at SODS] book appointments for me” (P010)*, while another mentioned “*they are making a link or connection with other doctors, which was good for me.” (P006)*

*Settlement* counsellors play a vital role as mediators between access and positive care perceptions as voiced by one participant *“when I first came to Canada…I was usually busy at work, and Global Gathering Place used to contact my family, and they used to take them to the clinic.”(P013)*

### Clinic Impact on Perceived Health Status

Table 3 shows that most respondents described their overall general health as either excellent or good (51% and 30% respectively). One participant explained that doctors in transit countries spoke to her as if there was no way to help her. After interacting with the REACH clinic doctors, she felt hopeful about life and her health “*it’s different when I came here, I’m having treatment and it’s different. Yes, I’m so happy; I’m positive.” (P004) A*fter visiting the clinic during the first year, 57% reported an improvement in their health status, while 33% (24/72) felt no change in their health and 10% perceived their health got worse. Improved health was attributed to the care provided by the clinic staff. *“I have blood pressure issues, [and] after using the clinic I feel better, every week I have [a] check-up with [a] doctor [and] the doctor was consistent and gave me medication” (P001)*

**Table 3:** Participants self-reported general health, quality of life, functioning and perceived health status

Survey questions:	Excellent n (%)	Good n (%)	Poor n (%)
Tell us about your general health today. (N = 73, missing 2)	37 (51)	22 (30)	14 (19)
Rate your quality of life (N = 70, missing 5)	42 (60)	27 (38)	2 (2)
Tell us about your health today. (N = 73, missing 2) Mood Sleep Appetite	32 (44) 39 (53) 46 (63)	26 (36) 21 (29) 21 (29)	15 (20) 13 (18) 06 (8)
Compared to one year ago, how would you say your health is now? (N = 72, missing 3)	Positive change = 41 (57%) No change = 24 (33%) Negative change = 7 (10%)		

After controlling for factors such as age and gender, multivariable analysis, as shown in Table 4, found that participants who received advice from doctors on how to stay healthy had 5.78 times odds of reporting a positive health status than those who did not report receiving such advice. Similarly, those who visited the clinic less frequently (≤ 4 visits) had 6.23 times odds of reporting a positive outcome compared to frequent users (p-value ≤ 0.05). 

**Table 4:** Variables associated with positive change in health status, the p-values, OR and 95% Cl

Variables	p-value	OR	95% CI
Age (24–44)	0.872	0.85	0.12–5.92
Age (45+)	0.812	0.78	0.10–5.98
Gender (male)	0.931	1.06	1.27–1.39
Education (primary)	0.161	3.08	0.28-4.00
**Frequency of visits (less than or equal to 4)**	**0.042**	**6.23**	**1.07–36.27**
Interpretation always available	0.432	0.55	0.12–2.46
Appointment availability	0.729	1.33	0.268–6.57
**Doctor gave advice on how to stay healthy**	**0.039**	**5.78**	**1.09–30.61**
Information explained	0.296	0.45	0.10–2.02
Doctor listened to my concerns	0.193	8.97	0.33-243.37

*p < 0.05 is statistically significant

### Refugees’ Experience After leaving REACH- Qualitative Findings

Three salient themes emerged as participants shared their experiences transitioning from the REACH clinic to mainstream care.

### Feeling Vulnerable and Powerless

Participants felt confused and unsure of the process, which led to a negative perception of the transition. They knew how the clinic operated and what services were available; they were comfortable and had very few difficulties at the clinic. A few participants felt vulnerable and powerless during the transition because of their unfamiliarity with the framework and structure of the healthcare system. As individuals coming out of precarious situations where they felt they had no rights or privileges, being new to this society adds another layer of uncertainty, fear, and vulnerability. This subtheme was implicit in the responses of participants. Even though the participants did not feel ready to leave the clinic, they thought they had no choice. The participants shared that they felt they had to respect the rules/laws of the clinic if they wanted to stay in Saskatoon or even have continued access to health care in general.

An unprepared mother expressed concerns about her inability to speak English and the possible repercussions that this lack of fluency might have in other clinical settings where there are no interpreters present. A mother caring for her newborn echoed similar concerns,

*“I really, really wanted to stay there [at the clinic], but I didn’t have a choice; it’s one year, then I have to leave.”* (P007)

One university-educated participant offered the following feedback:

*“It’s like you have to follow the rule, that’s the rule it’s only one year, so you have to look for another doctor. It was a little strange because when you got used to one doctor and then to another one, it’s like they disturb you, it’s like you’re not comfortable.”* (P006)

These comments suggest that removing an unprepared patient from the clinic and transitioning them to a family physician within 12 months of settlement might create a heightened sense of fear and vulnerability, which could result in feelings of re-victimization.

#### Experiences with a New Physician

A few participants expressed disappointment with the perceived inappropriate *matching* with community physicians who did not speak their language. Issues with health literacy, the absence of interpreters at the new physician’s office, and limited English language proficiency interfered with most refugees’ ability to maintain positive health-seeking behaviors. Unfamiliarity with the service delivery model utilized in mainstream care also created unexpected challenges.

*“I went to see a doctor, and they started to tell me my issue, but the doctor [told] me after two weeks, ‘I wanna see you’. But I don’t understand what the doctor is trying to say, so in two weeks I forgot, and they will send a bill I have to pay. I didn’t show up when the appointment that you were supposed to come. So, because of English, it is very hard for me to understand or remember when the appointment will be.”* (P001)

In contrast, participants who spoke the same language as their new physicians or those with greater English proficiency had better experiences adjusting to the new service delivery model and their new physician. A concerned husband whose wife had to change physicians three times recalled the difficulties his wife experienced before finding an appropriate doctor who spoke Arabic. He said:

*“My wife has lots of issues [female health issues], but she wasn’t confident to talk with other [doctor] because of language… but this doctor, she is really comfortable. And the good point is that she speaks Arabic, so she’s comfortable to like share [her] issues.”* (P008)

Additionally, participants felt the new family physician needed to be more knowledgeable, caring, and helpful. These perceptions prevented some physicians from establishing effective interpersonal relationships with patients and interfered with the effective continuity of patient care. Participant P001 said:

*“Some doctor you’re going to see, and they’re going to work on you, you’re going to feel comfortable, you’re going to feel good. But for the doctor that they have, they just go there, and it’s, ‘Why are you here? Tell me the things that bring you here; you’re going to feel [the doctor does] not care, and I do not even feel comfortable discussing with him [my health problem].”*

#### The Gap Between Expectations of Care and Doctor’s Delivery of Care

One father sought out care from a doctor of similar ethnicity for his daughters after recognizing the difference in cultural beliefs between his family and their assigned family doctor. The disappointed father shared that the doctor had over-stepped his responsibilities, asking his daughter about boyfriends and sex. He perceived the doctor to infringe on his cultural and traditional beliefs, and parental rights. He felt that the doctor did not respect their way of life.

*“My daughter is 15 to 16, and she [the doctor] talk[s] with her something about boyfriend, sex [the daughter visited the doctor with her mother, who does not speak English], I’m surprised, why [does] she [has] to talk about this. This is not her job, I’m so angry and not happy, and I told her I will move our file to another doctor.”(P008)*

Finally, participants mentioned that community doctors had little time to care for their various illnesses and provide follow-up. At the REACH Clinic, participants enjoyed the time spent with the doctors and the constant follow-up by both providers and staff. This indicated best practice, and it became an expectation of care. However, doctors in the mainstream healthcare system have greater patient loads with little support.

*“There is one time he went to see the [new] family doctor, and he forgot something; he was already outside, so he tried to come back to tell the family doctor what he forgot, but the family doctor was like, I don’t have time." *(P002)

## Discussion

The findings reveal that the clinic plays a welcome gatekeeping role in allowing refugees to easily access primary and tertiary care services [15]. Coordinating care by grouping several specialized services such as pharmacy, laboratory, referrals, interpreters, physicians who are not limited by systemic time pressure, and involving settlement agencies into the circle of care created a positive perception of quality care and gave them a sense of security. As reported by previous studies [7, 16, 17], the success of integrated refugees’ health care access while responding to their social needs has been shown to improve refugees’ satisfaction and quality of life [9]. The study also highlights that refugees who perceived their health status positively may have used the clinic services less frequently because they were likely healthier and had greater self-reported quality of life, possibly because of their younger age or ability to manage their existing health problems more effectively [18]. This implies a possible connection between frequent visitors and deteriorating health conditions or multiple comorbidities that required multiple follow-up visits. Many frequent visitors (over 5 visits) were females of child-bearing age likely seeking prenatal care or care for their children.

The quality of doctor-patient relationship and settlement agencies’ involvement are great mediators of refugees’ perceived positive health status. Those who received advice from physicians about ways to stay healthy were significantly more likely to report a positive health status after one year. For some refugees, health and illness are deeply linked with traditional beliefs and their perceptions of physicians [19, 20]. Physicians at REACH sought to establish a trust relationship to facilitate positive health outcomes. They used non-verbal cues, active listening, being respectful and kind, offering personalized and patient-centred care to better refugees’ experience. These findings echo prior studies that have underscored the impact of positive patient-physician interactions and personalized care that is not restricted to ethnicity, race, or language [21–24].

The study findings about interpretation corroborated evidence reported in the literature about the way interpretation services improve patient satisfaction [25, 26]. Participants described the ease of communicating with staff and physicians at REACH, in contrast with their current difficulties of speaking a language different from their new primary care doctor. At the time of transition to a community family physician, many refugees had not yet become proficient in English. Because of the language discordance, they were struggling to reach a level of comfort and trust with their newly assigned community primary care physician [27, 28]. Evidence emphasizes the need for greater language support such as a funded telephone interpretation service accessible to all providers in the community [29]. Currently, in Saskatchewan, telephonic interpretation services, such as CanTalk, are provided only in hospitals [29]. Integrating language interpretation services into healthcare systems (private and public) is vital in addressing the issues associated with language discordance between physicians and vulnerable groups, particularly ethnic minorities.

The positive experiences at the REACH clinic created great expectations about mainstream care. The REACH integrated model connected refugees with real-time access to care through a well-coordinated program. However, this may have complicated the transition to mainstream care, where the fee-for-service model dictates a different care delivery and may disenfranchise the most vulnerable groups from appropriate quality care [15]. From the perspective of community physicians, many challenges exist at the systemic and individual levels that prevent physicians from optimizing refugee care within the community. Physicians’ challenges include communication and cultural barriers, shortage of health care providers and services, and refugees’ medical background [30, 31]. When considering the many factors reported by the respondents that affect refugee transition to mainstream care, specialized clinics should ensure that conditions to successfully move to a community physician are met [32]. The “Beacon Model” [33] funded by Nova Scotia Health focuses on assessing refugees’ readiness level for transition to care in the community and supports capacity building activities for community providers, primary care physicians and provincial partners. This “Beacon Model” provides refugees with the support to access community providers, and to become more autonomous. As reported by Lane et al., refugees’ capacity for resourcefulness and self-reliance occurs when the right conditions are met [6]. Subsequent to this evaluation, the REACH clinic is working on improving refugees’ health literacy and knowledge about the health care system, as well as a transition model based on patients’ readiness rather than just time in the program.

Reliance on volunteers to help with interpretation when completing questionnaires and interviews may have biased some of the participants’ answers. Selection bias based on clinic access and program adherence may have also limited our findings. We used a self-reported questionnaire to capture refugees’ views and perspectives; however, biases arising from self-reporting, such as social desirability and selective recall, may have limited our interpretation of the results. Some husbands were also present when their wives were a respondent. However, access to interpreters allowed the inclusion of a diversity of respondents, such as those with little formal education.

With such diverse cultural and linguistic population residing in Saskatoon and across Canada, a more robust, innovative, and publicly funded service delivery model that includes healthcare navigators would provide additional support to accompany refugees and their families as they join mainstream care. Specialized refugee clinics should increase their collaboration with settlement agencies to augment the likelihood of successful transitions, which would include health literacy education and an assessment of patient’s readiness to move to community care. However, these steps require upstream funding and more extensive government partnerships. Similarly, community physicians require ongoing support, including funded interpretation services. Providing practising physicians with more robust support through Continuing Medical Education opportunities would increase the uptake of current practice guidelines in enhancing communication and delivering personalized, culturally safe care to refugees.

### New Contribution to the Literature

This study attempted to give voice to refugees about their perceptions of care and self-reported health outcomes while being provided an integrated model of care. Findings also highlighted that moving from such protected care setting to community-based care were met with a variety of reactions and emotions depending on preparedness factors such as familiarity with the healthcare system and linguistic concordance. Targeted funding for cultural navigators, easy access to interpretation and culturally safe training for health care professionals are three key recommendations that warrant further research.

## Footnotes

1 The Saskatoon Refugee Health Collaborative network comprise representatives from Global Gathering Place (GGP); Saskatoon Open Door Society (SODS); Saskatoon Community Clinic; Saskatchewan Health Authority formerly Saskatoon Health Region, Mental health and Public Health; and the University of Saskatchewan (Department of Paediatrics, Family Medicine and Community Health and Epidemiology).

2 (IBM Corp. Released 2010. IBM SPSS Statistics for Windows, Version 19. Armonk, NY: IBM Corp).
